# Reproducibility and geometric accuracy of the fixster system during hypofractionated stereotactic radiotherapy

**DOI:** 10.1186/1748-717X-3-16

**Published:** 2008-05-28

**Authors:** Peter Lindvall, Per Bergström, Per-Olov Löfroth, Roger Henriksson, A Tommy Bergenheim

**Affiliations:** 1 Department of Neurosurgery, Umeå University Hospital, Umeå, Sweden; 2 Department of Radiation sciences, Umeå University Hospital, Umeå, Sweden

## Abstract

**Background:**

Hypofractionated radiotherapy has been used for the treatment of AVMs and brain metastases. Hypofractionation necessitates the use of a relocatable stereotactic frame that has to be applied on several occasions. The stereotactic frame needs to have a high degree of reproducibility, and patient positioning is crucial to achieve a high accuracy of the treatment.

**Methods:**

In this study we have, by radiological means, evaluated the reproducibility of the isocenter in consecutive treatment sessions using the Fixster frame. Deviations in the X, Y and Z-axis were measured in 10 patients treated with hypofractionated radiotherapy.

**Results:**

The mean deviation in the X-axis was 0.4 mm (range -2.1 – 2.1, median 0.7 mm) and in the Y-axis -0.3 mm (range -1.4 – 0.7, median -0.2 mm). The mean deviation in the Z-axis was -0.6 (range -1.4 – 1.4, median 0.0 mm).

**Conclusion:**

There is a high degree of reproducibility of the isocenter during successive treatment sessions with HCSRT using the Fixster frame for stereotactic targeting. The high reducibility enables a safe treatment using hypofractionated stereotactic radiotherapy.

## Background

Hypofractionated stereotactic radiotherapy (HCSRT) is a method of delivering stereotactic irradiation in a few fractions using a relocatable stereotactic frame. This treatment is currently used for the treatment of arteriovenous malformations (AVMs) [1-4] and brain metastases [5,6]. HCSRT may be more appropriate than single fraction radiosurgery (SRS) for the treatment of large lesions or lesions located in eloquent areas. HCSRT enables the delivery of a higher total dose than possible with SRS without an increased risk of radionecrosis [1]. Fractionated stereotactic radiotherapy may also provide a radiobiological advantage over SRS in the treatment of malignant tumours [7]. HCSRT has been used for the treatment of AVMs and single or oligo brain metastases since 1986 at Umeå university Hospital. Results in terms of obliteration of AVMs has been evaluated and found to be comparable with SRS even though our AVMs were larger than in most series with SRS [1]. The standard treatment schedule for AVMs is 35 Gy in 5 fractions and for brain metastases 40 Gy in 5 fractions. The dose was normalized and specified to the center of the target and the 90% isodose line always encompassed the planning target volume. The procedure of hypofractionation and the relocatable stereotactic frame used for AVMs has been described earlier [1]. In order to deliver a hypofractionated treatment it is necessary to use a relocatable stereotactic frame. The relocatable Fixster frame [8,9] has been used by us for the treatment of brain metastases [6]. The accuracy of the stereotactic treatment will among other factors depend on the reproducibility of the stereotactic frame and the positioning of the patient. It is necessary that the frame and the patient can be positioned in the exact same way for each treatment session in order to deliver the irradiation according to the dose plan. Other stereotactic frames used for fractionated radiotherapy are the Laitinen stereoadapter (LS) and the Gill-Thomas-Cosman frame (GTC). These frames have reported a high level of reproducibility with a geometrical accuracy of less than 1 mm for the LS [10,11] and a overall accuracy of 1.7 ± 0.7 mm for the GTC [12]. In the case of the Fixster system there is no study that has investigated the accuracy of the frame regarding reproducibility in a clinical treatment situation. The Fixster head fixation system was first described by Greitz et al., and in the original paper it was reported to have a maximum deviation of 2–3 mm in terms of reproducibility of the frame [9]. According to Bergström et al., the accuracy for coordinate determinations in a phantom had a maximum error of 1 mm [8]. In this study we have evaluated the clinical reproducibility of the total set up procedure in consecutive treatment sessions of patients with brain metastases using the relocatable Fixster frame.

## Methods

**Figure 1:**
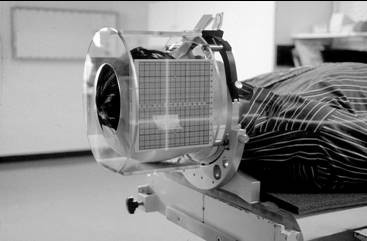
Patient in a treatment situation, the Fixster frame applied and infrared beams indicating the isocenter.

**Figure 2:**
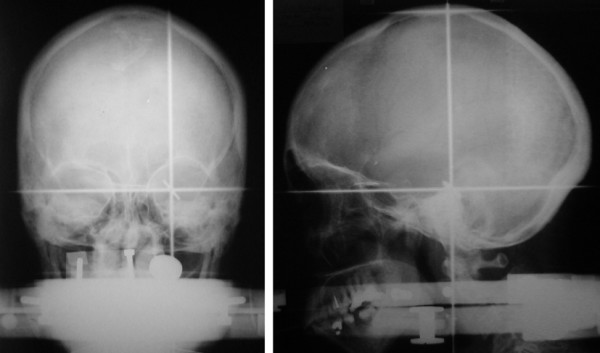
Orthogonal plain X-ray images; lateral and anterioposterior views.

Ten patients diagnosed with cerebral metastases were treated with HCSRT using the Fixster frame for stereotactic targeting of the lesion in every treatment session. The local ethical committee at the Umeå University Hospital approved this study, and all patients had given an informed consent in participating in this study. Before treatment a stereotactic CT examination with the Fixster frame was performed in all patients for doseplanning [6]. During treatment the patients were positioned on the coach of a Linear accelerator (Varian 2300 C/D). The rotation center of the linear accelerator was positioned in the isocenter of the dose plan by alignment of the calibrated narrow laser cross lines in the treatment room to marked positions on the side plates of the frame (Fig 1). A careful and precise test of reproducibility was not possible to perform in the treatment room, and was therefore performed at the simulator where an X-ray facility was available (the Oldelft MC). After each of three consecutive treatment sessions the patients had the Fixster frame carefully applied and positioned in the simulator room. Indicators were mounted on the side plates of the frame to facilitate the evaluation. Two orthogonal plain X-ray images; lateral and anterioposterior views (Fig. 2), were taken with the Fixster frame in position. The first set of X-ray images was used as a template, and the center of the target was carefully marked. A pencil was used to mark the inner table of the skull bone and bone landmarks on the lateral and anterioposterior views; the orbital rim, the sphenoid sinus and the sella. Images from the next two investigations were marked in the same way and superimposed on the corresponding projection. The deviation in X, Y, and Z from the isocenter on the original investigation was measured and corrected with the magnification factor on the X-ray images to achieve the real deviation. Deviation to the right in the X-axis, laterality, was assigned positive values and to the left negative values. Deviation in the frontal direction in the Y-axis, anterio-posteriorly, was assigned a positive value and a deviation the opposite direction a negative value. Finally, in the Z-axis, cranio-caudal, deviation caudally towards the skull base was assigned a positive value and deviation in the cranial direction was assigned a negative value.

## Results

**Figure 3:**
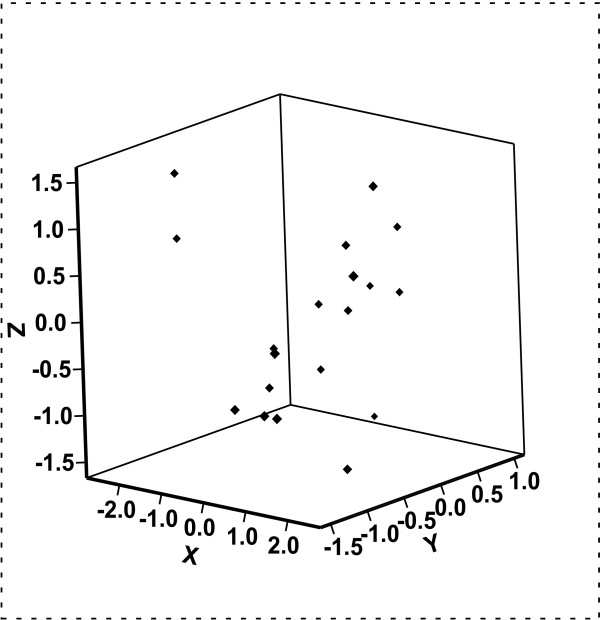
Three dimensional graph showing deviations in the X, Y and Z-axis.

The deviations in the X, Y and Z-axis are shown in Table 1 and Fig. 3. The mean deviation in the X-axis was 0.4 mm, (range, -2.1 – 2.1, median, 0.7 mm) and in the Y-axis -0.3 mm (range, -1.4 – 0.7, median, -0.2 mm). The mean deviation in the Z-axis was -0.6 mm (range, -1.4 – 1.4, median, 0.0 mm).

**Table 1:** Deviation in the X, Y and Z axis.

**Patients**	**Dev X (mm)**	**Dev Y (mm)**	**Dev Z (mm)**
1	-0.4	-0.4	-0.4
	0.7	0.7	0.7
2	0.7	0.0	0.0
	1.4	0.0	1.4
3	1.4	-1.4	0.0
	0.0	0.7	-1.4
4	0.4	-1.4	-0.7
	2.1	-0.7	0.7
5	-2.1	-0.7	0.7
	0.0	-0.7	-0.7
6	1.4	-1.4	-0.7
	1.8	-0.7	-1.4
7	-2.1	-0.7	1.4
	1.1	-1.4	-0.7
8	0.7	0.7	0.0
	0.7	0.0	0.7
9	0.7	0.7	0.0
	0.0	0.7	0.0
10	0.0	0.0	0.0
	0.0	0.0	-0.7

## Discussion

There seems to be a high degree of reproducibility of the isocenter after repetitive positioning of the Fixster frame during treatment sessions with HCSRT. The largest deviation was observed in the X-axis with a maximum deviation of 2.1 mm at one occasion. The high accuracy and precision of SRS as an alternative to HCSRT has previously been documented [13]. Even simulation of a multistage treatment in a phantom using SRS shows a high accuracy with a maximum error of 1 mm after sequential placement of the Leksell stereotactic head frame [14]. There has been an increased interest in HCSRT for the treatment of brain metastases and AVMs as an alternative to SRS [3-5]. Treatment with HCSRT may allow the delivery of a higher total dose than possible with SRS. There might be concern that fractionation with a non-invasive relocatable stereotactic frame and patient positioning for treatment may compromise the precision of the treatment. In our treatment of brain metastases we use a stereotactic frame that has been described in previous publications. The Fixster frame may also be used for other purposes such as treatment of non-operable skull base meningeomas. At our departments, however, we do not use a hypofractionated schedule for this treatment due to the often close relationship to eloquent structures including the optic nerve. In these cases irradiation is delivered in 2 Gy fractions to a total dose of 56 Gy. In our study deviations in the three dimensions (X, Y and Z) are not solely a measurement of the precision and reproducibility of the stereotactic frame but include also the set up alignment for repeated treatment sessions. Thus we have measured the maximum deviation of the isocenter during successive simulated treatment sessions. We believe that this is a more accurate way to evaluate the precision in the treatment than to only evaluate the reproducibility of the stereotactic frame itself.

The two most commonly used relocatable non-invasive stereotactic frames used for fractionated radiotherapy are the LS and the GTC. The reproducibility of the LS in patient studies has proved to be less than 1 mm [10,11,15]. The GTC frame has in two recent studies shown a reproducibility with a mean error of 1.7 and 1.8 mm [12,16]. The reproducibility and accuracy of the Fixster frame in a clinical treatment situation has not been described previously. The maximum deviations after successive mountings of the Fixster frame, including patient positioning before treatment, seem to be in the range of what has been reported for the other relocatable non-invasive frames used for fractionated radiotherapy. Even in the case of a maximum error the targets should be covered by the margin added to generate the planning target volume. A 2 mm margin is added to the nidus for AVMs, and a 3 mm margin for brain metastases. There is of course a risk that the positioning of the patient will be more carefully done during an investigational assessment than during routine treatment. However, using a non-invasive stereotactic system one has always to be aware of this issue and at all occasions be meticulous when positioning the patient.

## Conclusion

There is a high degree of reproducibility in successive treatment sessions with HCSRT using the Fixster frame for stereotactic targeting. The isocenter show only a small deviation in the X, Y and Z-axis after consecutive treatment sessions including repetitive mounting of the Fixster frame and patient positioning. Thus, hypofractionated stereotactic radiotherapy using the non-invasive relocatable Fixster frame shows a high accuracy despite the need for repetitive application of a stereotactic frame and patient positioning.

## Competing interests

The authors declare that they have no competing interests.

## Authors' contributions

PL responsible for the study design, data analysis and writing of the manuscript.

PB/POL involved in the design of the study and acquisition of data.

RH/ATB study design, analysis of data and results, and finally in the writing of the manuscript

All authors have read and approved the final version of the manuscript.
